# Impact of chronic exposure to the pesticide chlorpyrifos on respiratory parameters and sleep apnea in juvenile and adult rats

**DOI:** 10.1371/journal.pone.0191237

**Published:** 2018-01-22

**Authors:** Walaa Darwiche, Jérôme Gay-Quéheillard, Stéphane Delanaud, Hiba El Khayat El Sabbouri, Hassan Khachfe, Wissam Joumaa, Véronique Bach, Wiam Ramadan

**Affiliations:** 1 PériTox, Périnatalité & Risques Toxiques, UMR-I 01 INERIS, Amiens, France; 2 PhyToxE, Environmental Physio-Toxicity group, Rammal Hassan Rammal laboratory, Lebanese University, Faculty of Sciences, Nabatieh, Lebanon; 3 Lebanese Institute for Biomedical Research and Application (LIBRA), Lebanese International University (LIU), Beirut, Lebanon; Weizmann Institute of Science, ISRAEL

## Abstract

The widely used organophosphorus pesticide chlorpyrifos (CPF) is often detected in food. CPF inhibits acetylcholinesterase and can modify muscle contractility and respiratory patterns. We studied the effects of chronic exposure to CPF on respiratory parameters and diaphragm contractility in 21- and 60-days old rats. Pregnant rats were exposed to oral CPF (1 or 5 mg/ kg /day: CPF-1 or CPF-5 groups vs vehicle: controls) from gestation onset up to weaning of the pups that were individually gavaged (CPF or vehicle) thereafter. Two developmental time points were studied: weaning (day 21) and adulthood (day 60). Whole-body plethysmography was used to score breathing patterns and apnea index during sleep. Then, diaphragm strips were dissected for the assessment of contractility and acetylcholinesterase activity. Results showed that the sleep apnea index was higher in CPF-exposed rats than in controls. In adult rats, the expiratory time and tidal volume were higher in CPF-exposed animals than in controls. At both ages, the diaphragm’s amplitude of contraction and fatigability index were higher in the CPF-5 group, due to lower acetylcholinesterase activity. We conclude that chronic exposure to CPF is associated with higher sleep apnea index and diaphragm contractility, and modifies respiratory patterns in sleeping juvenile and adult rats.

## Introduction

Organophosphorus pesticides (OPs) are commonly used in agricultural, industrial and domestic settings [1]. Residues are found not only in indoor and outdoor environments [2] but also in food and drinking water [3]. Exposure to OPs occurs mainly via dietary intake in adults [4] and children [5]. Various metabolites of OPs have been found in meconium samples from newborns having been exposed during pregnancy [6]. After birth, newborns were further exposed via breastfeeding [7].

OPs are potent acetylcholinesterase (AChE) inhibitors, and thus produce excessive levels of neurotransmitter at cholinergic synapses [1]. Since acetylcholine (ACh) is an essential neurotransmitter in the regulation of respiratory parameters (especially with regard to the conduction of afferent signals from the chemoreceptors in hypercapnia and hypoxia [8]), impaired synaptic transmission may have drastic effects on the control of these physiological processes. After acute exposure to OPs, elevated ACh levels at the motor end plate lead to cholinergic hyperstimulation, bronchoconstriction, and depression of the respiratory centers in the brainstem [9]. Central apnea is a feature of acute OP intoxication, and may even lead to death after exposure in humans [10]. Although the effects of acute exposure are well known [11][12], the effects of chronic exposure to lower doses of OPs on the respiratory system have not been extensively characterized.

The widely used OP chlorpyrifos (CPF, O,O-diethyl-O-[3,5,6-trichloro-2-pyridinyl] phosphorothioate) is metabolized in humans to CPF-oxon, an active metabolite that also acts as a potent anti-cholinesterase [13]. Juvenile rats have lower activities of CPF-detoxifying enzymes than adult rats [14] and thus are more vulnerable to CPF toxicity [15]. CPF residues can be detected in milk [7] and cord blood samples [16]. Exposure to CPF during gestation interferes with the development of the brain [17] by disrupting neural cell replication and differentiation, axonogenesis, and synaptogenesis in regions innervated by cholinergic projections (such as the respiratory centers) [18]. Thus, one can legitimately hypothesize that chronic exposure to CPF could induce respiratory disorders in juvenile and young adult rats.

To the best of our knowledge, the effects of a chronic life-time exposure to OPs (and to CPF in particular) on respiratory parameters have never previously been assessed. The objective of the present study was to analyze the effects of chronic CPF exposure on 21-day-old rats (juveniles) and 60-day-old rats (young adults). Measurements were made both in vivo (respiratory parameters and sleep apnea, using whole-body plethysmography) and in vitro (AChE activity and contractility of the diaphragm).

## Material and methods

### Chlorpyrifos preparation

Chlorpyrifos (O,O-diethyl-O-(3,5,6-trichloro-2-pyridinyl) phosphorothioate) with purity of 99.8% was purchased from LGC Standards (Molsheim, France). Chlorpyrifos was dissolved in commercially available rapeseed oil as vehicle and administered by gavage to the rats at 1 or 5 mg/ml (CPF-1 and CPF-5 groups, respectively). The dose 1mg/kg/day is the oral NOAEL for depression of brain acetylcholinesterase activity in rats (2 years study). The 5 mg/kg/day is above the NOAEL for developmental toxicity [19]. CPF solutions were maintained at room temperature protected from light. Animals in control groups were gavaged with the organic rapeseed oil as vehicle.

### Animals

The experimental protocol was approved by the Animal Care and Use Committee at Jules Verne University of Picardy (reference #2011/A/1, protocol number: 291112–19, Amiens, France). All animals were treated in accordance with the European Communities Council’s guidelines (2010/63/EU).

Three groups of 5 pregnant rats (Janvier Labs, Le Genest Saint Isle, France) were gavaged daily with organic rapeseed oil (as a control) or one of two doses of CPF (1 or 5 mg/kg/day in organic rapeseed oil, referred to as CPF-1 and CPF-5, respectively) from gestational day 1 until their pups were weaned (on post-natal day (PND) 21). The size of the litter was adjusted in order to obtain litters of the same size (8 pups) and only male rats were used in this study. Pups for each treatment group were obtained from a minimum of five litters. On PND21, the respiratory parameters for the half the pups (n = 10–12 per group) were measured via whole-body plethysmography. These animals were then euthanized with an intraperitoneal overdose of sodium pentobarbital (1 ml/kg; 200 mg/ml solution). The diaphragm was removed and rectangular strips were dissected from the midcostal region. The remaining rats (n = 10–12 per group) were gavaged with organic rapeseed oil (control), CPF-1 or CPF-5 until PND60. Respiratory parameters and the diaphragm’s contractile properties were then assessed using the procedures described above.

### Respiratory measurements

The inspiratory time (T_I_), expiratory time (T_E_), tidal volume (V_T_) (normalized to the body weight), and respiratory frequency (f) were obtained for each animal [20] using whole-body plethysmography (Model PLY3213, Buxco-EMKA Technologies, Sharon, CT). Before the respiratory measurements, rats were familiarized with the plethysmograph chamber twice for one hour. Apnea was defined as the cessation of ventilation for at least 2.5s (reflecting at least two missed breaths). The sleep apnea index (the number of apnea episodes per hour of behaviorally defined sleep) was scored using a previously validated procedure [21]. Measurements were made over a 60-minute period, during which the rat was continuously scored for wakefulness (i.e. lying or standing, with the eyes open) or sleep (i.e., lying down, not moving, with the eyes closed), as previously described [22]. Apnea episodes were excluded from the calculation of respiratory parameters.

### Diaphragm contractility and fatigue resistance

The diaphragm’s contractile properties were analyzed by studying rectangular strips dissected from the midcostal region. The strips were activated by electrical stimulation (12 V, 2 Hz) for 5 minutes. The twitch tension (g.cm^-2^), contraction time (ms), half relaxation time (ms), and fatigability index (%) were assessed as described elsewhere [23].

### Acetylcholinesterase (AChE) activity

AChE activity was measured according to Ellman’s method [24]. Diaphragm homogenates were incubated with 10^−5^ M of the butyryl cholinesterase inhibitor, tetraisopropyl pyrophosphoramide iso-OMPA (Sigma Aldrich, Saint Quentin Fallavier, France). A colorimetric AChE assay kit was used to measure the AChE activity according to manufacturer’s instructions (Abcam, Cambridge, UK) and the data were expressed in μmol/min/mU.

### Statistical analysis

Statistical analysis was performed using Prism 5 software (GraphPad Software Inc., La Jolla, CA). A two-way analysis of variance (ANOVA) was used to analyze the main effects of age (PND21/PND60) or exposure (Control/CPF-1/CPF-5) and the interaction between age and CPF exposure. If the age-CPF exposure interaction was significant, a one-way ANOVA and then an unpaired t-test were performed for the PND21 or PND60 group. All data passed statistical tests for normality and homogeneity of variance. The threshold for statistical significance was set to p≤0.05. Indicative results (p<0.1) are given when appropriate.

## Results

### Body weight of pups

**Fig 1:**
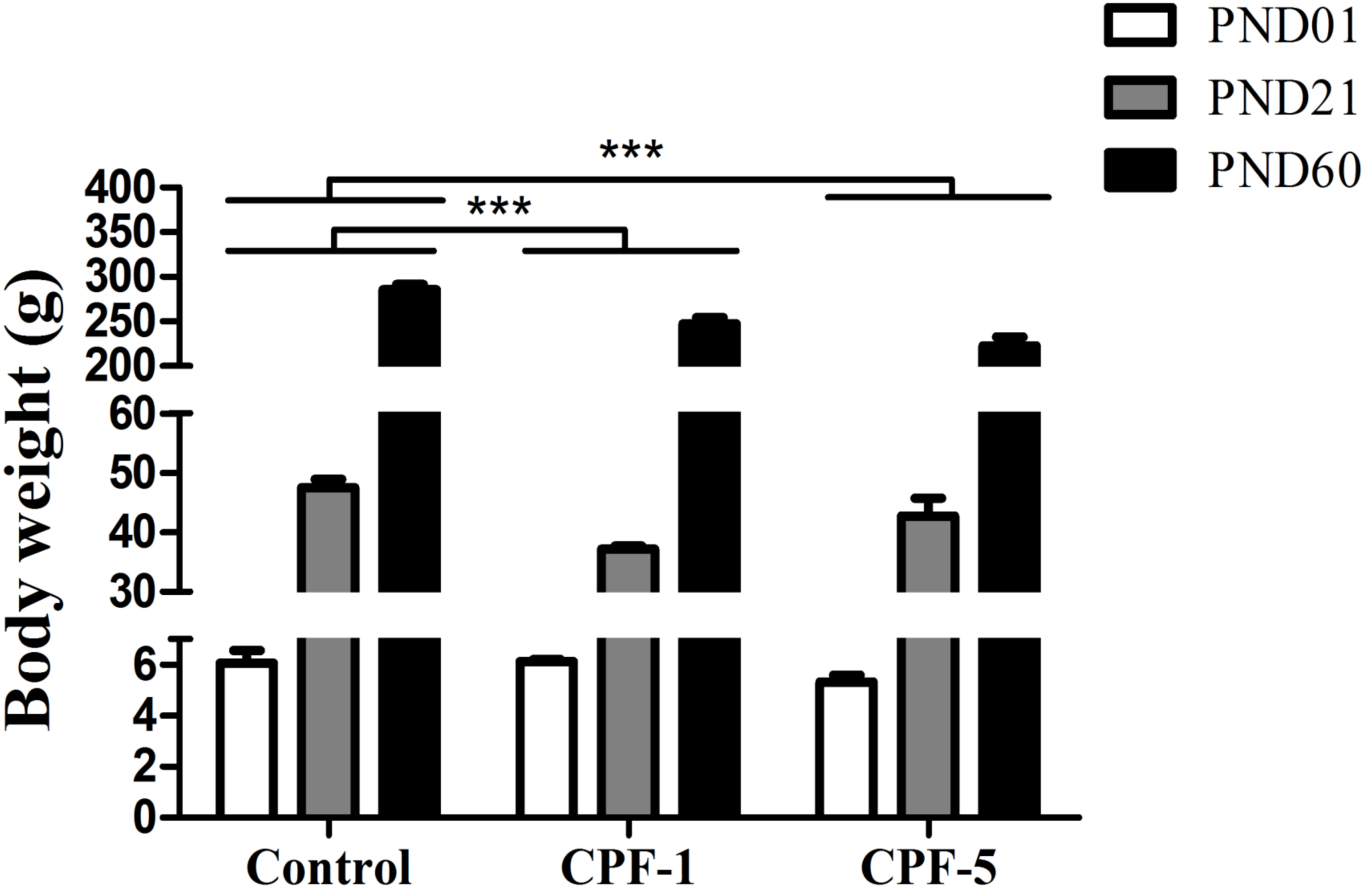
Effects of CPF exposure on the body weight of rat pups at birth (PND01, open bars), as juveniles (PND21, grey bars) and as young adults (PND60, black bars). Data are quoted as the mean ± SEM, at PND01 and PND21: n = 20-22/group; at PND60, n = 10-12/group. ***: p<0.001.

As shown in Fig 1, the mean bodyweight of rat pups exposed to CPF-1 and CPF-5 was significantly lower than that of the control group (p<0.001 for all).

### Respiratory parameters

**Fig 2:**
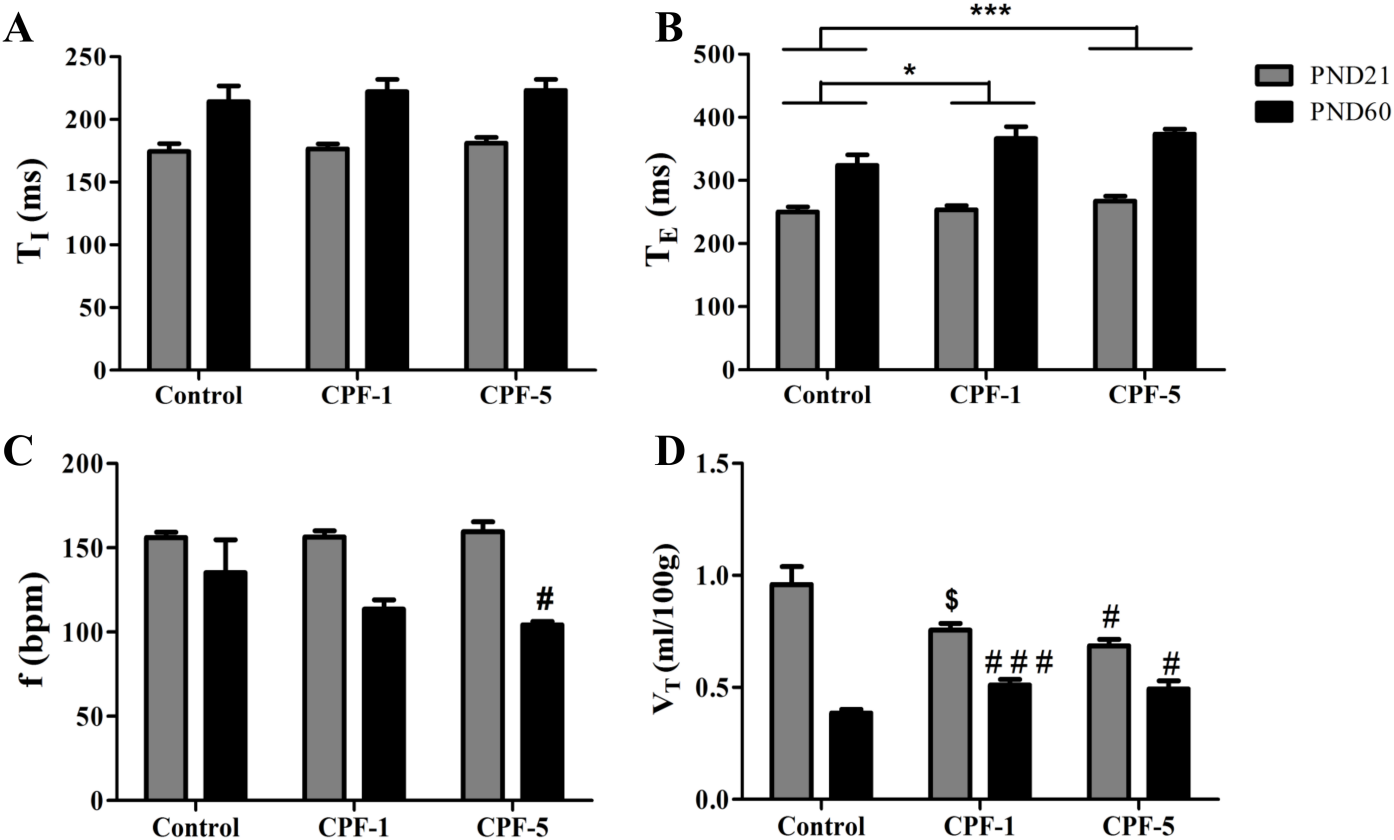
Effects of CPF exposure on respiratory parameters in juvenile rats (PND21 grey bars) and adult rats (PND60, black bars). (A) T_I_ (ms), (B) T_E_ (ms), (C) f (bpm), and (D) V_T_ (ml/100g body weight) in juvenile and adult rats. Data are quoted as the mean ± SEM, n = 10-12/group. Effect of CPF exposure: *: p<0.05; ***: p<0.001. For a significant age x exposure interaction: #: p<0.05; # # #: p<0.001; $: p<0.1 compared with controls of the same age (*unpaired t-test*).

Comparisons of the juvenile vs. adult rats evidenced significant changes over time in T_I_, T_E_, V_T_ and f (Fig 2), with a main effect of age (p<0.001 for all). T_I_ and T_E_ were higher in adults than in juvenile rats, whereas V_T_ and f were lower.

When we compared the exposed groups with the controls, we observed that the CPF exposure was associated with a significantly longer T_E_ at both doses (Fig 2B; *vs*. controls: CPF-1: p = 0.011, CPF-5: p<0.001). Furthermore, the effect on f of the age-exposure interaction tended towards significance (p = 0.07). The f was reduced by CPF exposure in the young adult group (the only significant difference was for control *vs*. CPF-5; p = 0.04) but not in the juvenile group (Fig 2C). A significant age-CPF exposure interaction was found for V_T_ (p<0.001). CPF exposure had different effects on juvenile rats vs. adult rats: at PND21, CPF exposure was associated with a lower V_T_ (*vs*. control: CPF-1: p = 0.059; CPF-5: p = 0.015). In contrast, at PND60, CPF exposure was associated with an elevated V_T_ (vs. control: CPF-1: p<0.001; CPF-5: p = 0.044) (Fig 2D).

**Fig 3:**
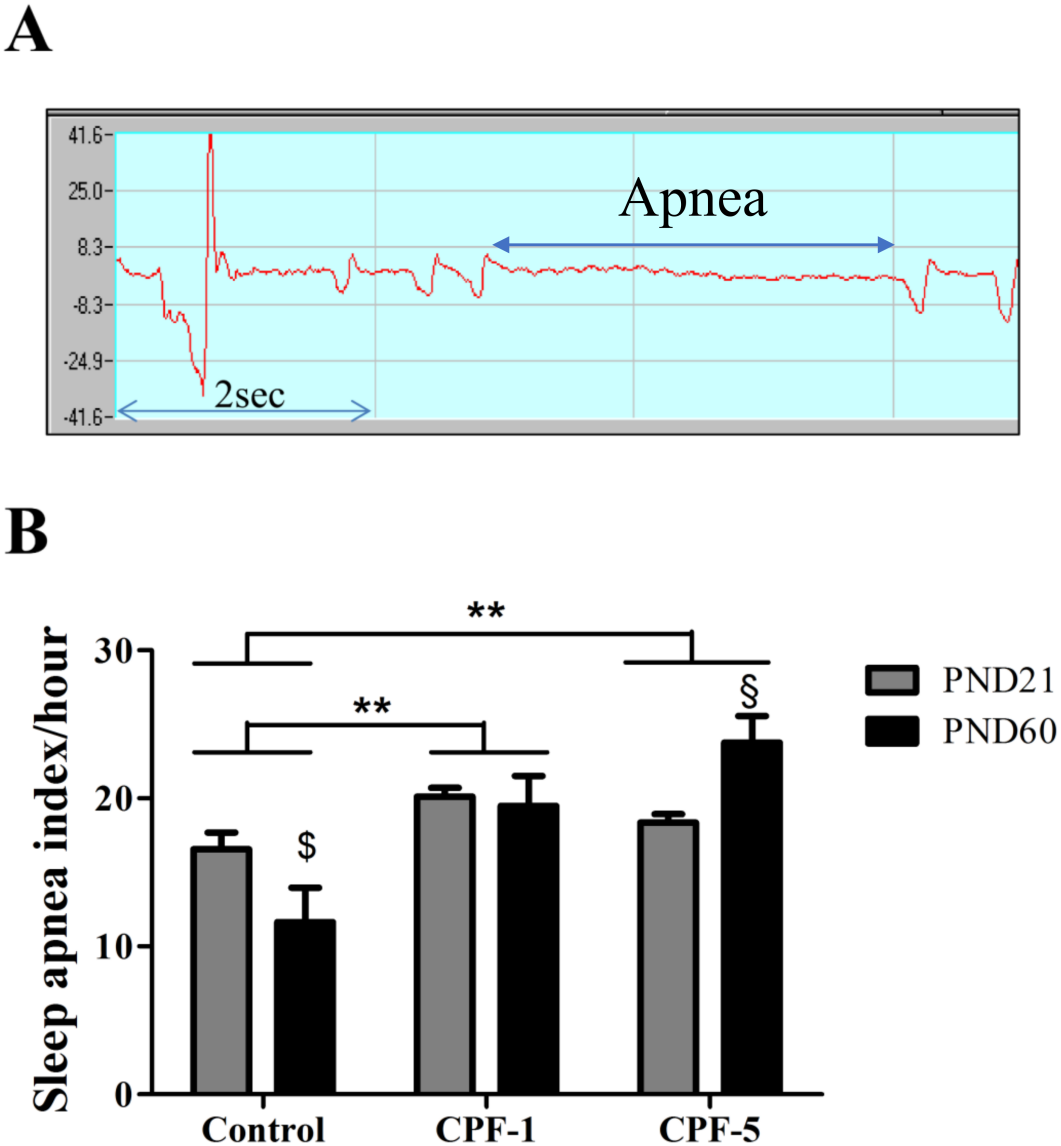
Effects of age and CPF exposure on the sleep apnea index in juvenile (PND21) and adult (PND60) rats. A) A representative plethysmographic signal corresponding to an episode of apnea in the rat lasting longer than 2.5 sec. B) Mean numbers of apnea episodes per hour in juvenile rats (PND21 grey bars) and adult rats (PND60, black bars) in the control, CPF-1 and CPF-5 groups. **: p<0.01. The age difference between PND21 and PND60 is shown: **§**: p<0.05, $: p<0.1 PND60 vs. PND21. Data are quoted as the mean ± SEM, n = 10-12/group.

As shown in Fig 3, both doses of CPF exposure were associated with a higher apnea index (*vs*. controls: CPF-1: p<0.01; CPF-5: p<0.01). A significant age-CPF exposure interaction was observed for the apnea index (p = 0.014). In the control (non-exposed) group, maturation from PND21 to PND60 tended to reduce the apnea index (p = 0.081). When exposed to the lower dose of CPF (CPF-1), the apnea index did not decrease with age, and thus remained similar in young adults and juvenile rats. In contrast, the apnea index in the CPF-5 group was significantly higher at PND60 than at PND21 (p = 0.03).

### Diaphragm contractility

**Fig 4:**
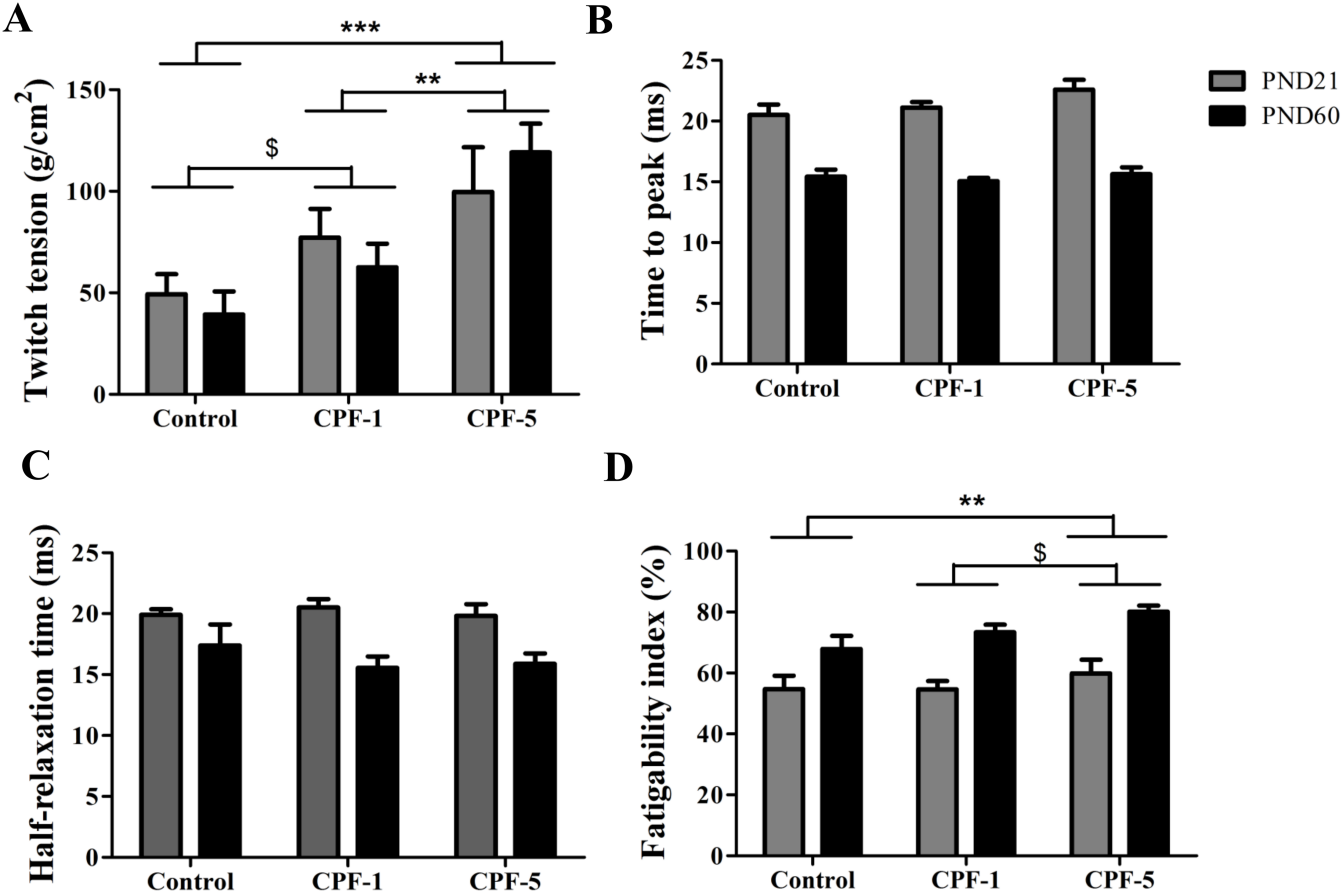
Effects of CPF exposure on diaphragm contractility in juvenile (PND21, grey bars) and adult (PND60, black bars) rats. (A) Twitch tension, (B) time to peak, (C) half relaxation time, (D) fatigability index. Effect of CPF exposure: **: p<0.01; ***: p<0.001, $: p<0.1. Data are quoted as the mean ± SEM, n = 10-12/group.

By comparing the diaphragm’s contractile properties in juvenile and adult rats, we found that the time to peak and the half relaxation time (Fig 4) were significantly lower in adults than in juveniles, whereas the fatigability index was significantly higher (p<0.001). There was no age-related difference in the diaphragm twitch tension.

A main effect of CPF exposure was observed for twitch tension (p<0.001), with a significant dose effect: the higher the dose of CPF, the higher the twitch tension (CPF-5 *vs*. control: p<0.001; CPF-1 *vs*. CPF-5: p<0.01; CPF-1 vs. control: p<0.1). The fatigability index tended to be higher in the CPF-exposed groups than in the control group (p = 0.056). This index was higher in CPF-5 group than in the controls and CPF-1 groups (*vs*. CPF-5: Control: p = 0.004; CPF-1: p = 0.067). In addition, the fatigability index tends to increase between CPF-1 and CPF-5 groups (p<0.1) (Fig 4D). In contrast, CPF exposure did not impact the time to peak and the half relaxation time (Fig 4B and 4C).

### AChE activity

**Fig 5:**
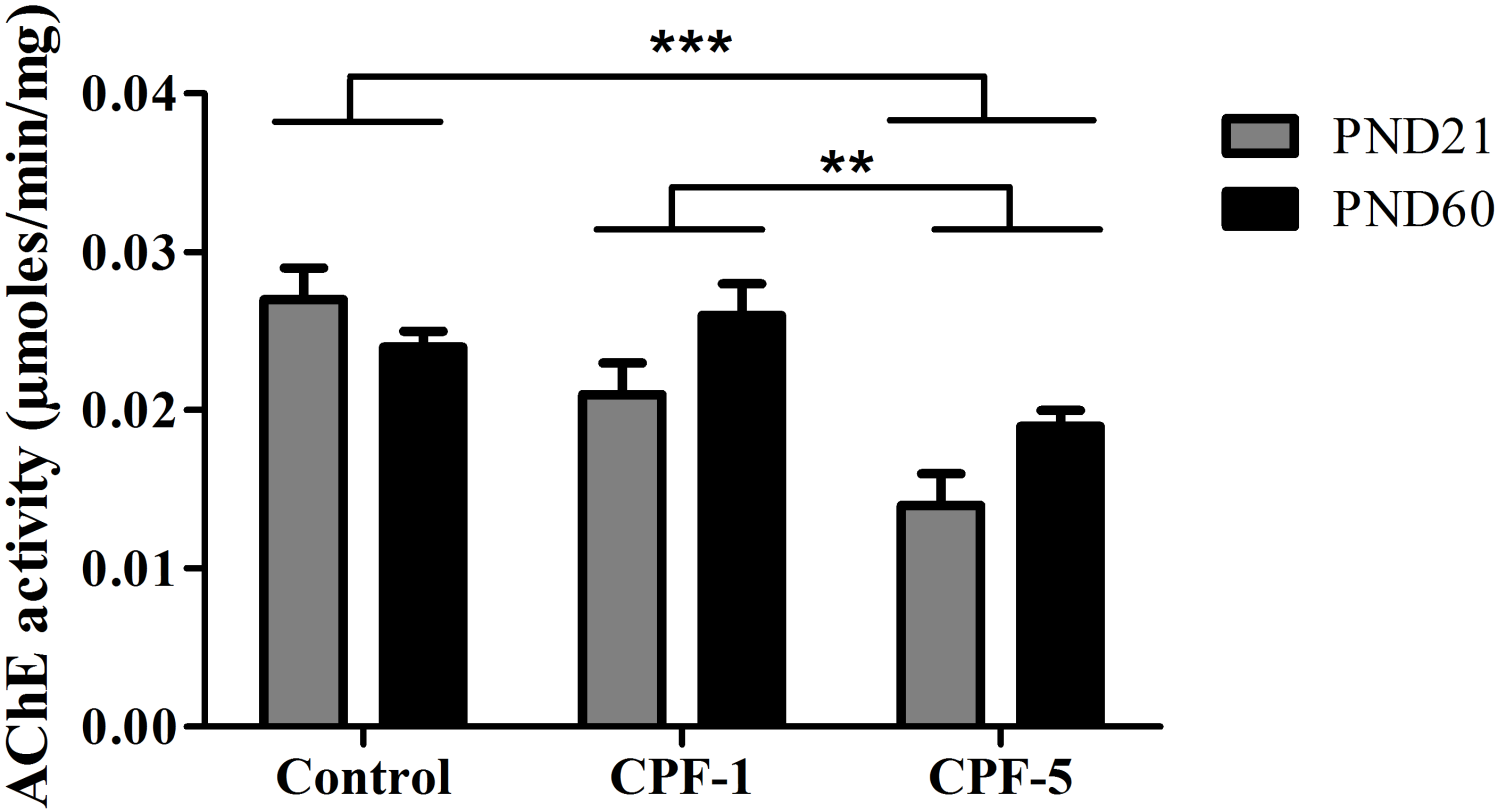
Effects of CPF exposure on AChE activity in the diaphragm in juvenile (PND21, grey bars) and adult (PND60, black bars) rats. **: p<0.01; ***: p<0.001 compared with controls or CPF-1. Data are quoted as the mean ± SEM, n = 10-12/group.

There was no age-related difference in AChE activity in the diaphragm. However, a dose-dependent variation in AChE activity was observed (control *vs*. CPF-5: p<0.001; CPF-1 *vs*. CPF-5: p<0.01) (Fig 5).

## Discussion

To the best of our knowledge, the present study is the first to have assessed the effects of chronic exposure to an OP on respiratory function in growing animals (juvenile and young adult rats). We assessed the respiratory parameters on juvenile (PND21) and adult (PND60) rats in order to show the effects of CPF exposure during pregnancy and breastfeeding period on juveniles (PND21) and the effects on young adults (PND60) if this exposure remains during post-weaning period. The sleep apnea index, T_E_ and V_T_ were higher in adult animals exposed to CPF than in non-exposed controls, whereas f was lower. The diaphragm’s amplitude of contraction and fatigability index were also higher in CPF-exposed groups, as a result of lower AChE activity. Furthermore, prenatal exposure of the rat pups to CPF was associated with low body weight at birth; this might be due to CPF’s toxicity. Importantly, the low body weight of CPF-exposed animals persisted at weaning (PND21) and into adulthood (PND60). Our results are in line with clinical cohort studies in which exposure to CPF adversely affected fetal development in infants whose mothers had been exposed to CPF during pregnancy and when breastfeeding [16][25]. Interestingly, low body weight is a known risk factor for an elevated apnea index, as a result of impaired neurologic maturation [26]. Furthermore, the rats in the present study were smaller (in terms of body length) than controls at birth (data not shown).

The effects of OPs on respiratory patterns have only previously been described during acute exposure [11][12][27]. It is well established that prenatal exposure to CPF impairs the development of the brain in general and the cholinergic areas involved in the regulation of breathing in particular [28][29][30].

The main finding of the present study is that chronic exposure of rats to CPF is associated with a change in respiratory patterns during sleep. T_E_ was longer in CPF-exposed rats. Consequently, the f tended to be lower in CPF-exposed groups. Our present findings are consistent with the documented effects of acute exposure to another OP (para-oxon) on respiration in animals [12]. Like other phosphate containing pesticides, CPF is metabolized to the corresponding oxygen analog (CPF-oxon) which phosphorylates and then inhibits the AChE activity in both central and peripheral nerve tissues [31], leading to an excess of acetylcholine. We measured the AChE activity in the brainstem of adult rats, we observed a significant decrease of AChE activity in the brainstem of rats exposed to 5 mg/kg/day of CPF (data not shown). As shown by Houze et al. [11] and Carey et al. [10], the effect of OPs on the respiratory pattern is due to overstimulation of the muscarinic receptors in the central nervous system. However, a peripheral action of CPF cannot be ruled out. Exposure to CPF is accompanied by bronchoconstriction and decreased muscarinic M2 receptor function after AChE inhibition [32]. A decrease in the upper airway diameter might explain the high T_E_, low f and high apnea index observed in the CPF groups.

CPF exposure was associated with a higher sleep apnea index (with a greater frequency but not a longer duration) in juvenile and adult rats, independently of the dose. Apnea arises through two mechanisms: (i) central depression of the respiratory centers and cessation of the respiratory command from the brainstem, and (ii) a peripheral effect of obstruction of the upper airways [33] and a decrease in the air flow, which increases respiratory work [34]. The well-characterized episodes of central apnea observed during acute exposure to OPs are caused by the inhibition of cholinesterase in the respiratory centers [10][34][35]. However, a peripheral trigger for apnea cannot be ruled out, since the OPs also result in airway obstruction [32] and greater total lung resistance with an increase in respiratory secretions [36]; this might also explain the longer T_E_ observed in our study. However, in our experimental setting, it is not possible to distinguish between central and peripheral apnea. Another important finding is that CPF exposure in the present study was associated with a higher apnea index in adult rats than in juvenile rats. In the control group, maturation from the juvenile age to adulthood tended to decrease the apnea index. It is well known that the occurrence of apnea becomes significantly less frequent with age and maturation [37]. However, when rats were exposed to CPF at the lower dose, the apnea index did not decrease with age. Moreover, the apnea index was higher when rats were exposed to the higher dose of CPF: there was a significant difference when comparing adult rats with juveniles in the CPF5 group. This observation indicates that CPF exposure might affects respiratory patterns by inhibiting the physiological processes through which apnea usually decreases with age. Nevertheless, the effects of CPF on respiration and sleep apnea at PND60 can be due to the (i) in utero and lactational exposure by the maternal diet (ii) CPF exposure after weaning or (iii) both. To distinguish between these possibilities, it would be necessary to study the respiration and sleep apnea at PND60 in a cohort of animals exposed to CPF only during gestation and lactation.

With regard to contraction of the diaphragm, there was a significant, dose-dependent, relative increase in twitch tension in the CPF groups at both ages. This increase in twitch tension was also reported by Burd and Ferry [38] after *in vitro* exposure to an anticholinesterase (echothiopate), as a result of a prolonged response to ACh at the neuromuscular junction. Wright et al. [39] reported an increase in phrenic nerve activity after exposure to an anticholinesterase. This increase is undoubtedly related to the low level of AChE activity observed in juvenile and adult rats exposed to CPF. The greater level of contraction might explain the increase in V_T_ in adult rats observed in our study (via an increase in the volume of the thoracic cage and thus an increase in the volume of inspired air). The greater level of contraction might also be related to the occurrence of apnea due to obstruction of the upper airways [9]; the diaphragm must contract more forcefully to overcome the obstruction-related resistance.

The present study highlighted greater diaphragm fatigability in juvenile and adult rats exposed to CPF5, relative to non-exposed animals. In fact, skeletal muscle weakness is one of the clinical effects observed after OP poisoning. It can be attributed to elevated ACh levels at the motor end plates (and thus overstimulation of nicotinic acetylcholine receptors) following AChE inhibition [40].

In conclusion, our present results evidenced the effects of (i) mother-to-offspring transmission of CPF and (ii) chronic oral exposure to CPF during early adulthood. The observed respiratory dysfunction could be resulted from changes in central and/or peripheral processes, due to the accumulation of acetylcholine in the synaptic clefts. However, it is not possible to rule out secondary alterations due to CPF effects on body weight. Chronic life-time exposure to CPF was associated with an elevated sleep apnea index, greater diaphragmatic contraction and thus greater fatigability. The present functional study of the rat diaphragm is the first to have highlighted the effects of chronic exposure to OPs on respiratory patterns in adulthood.

## Supporting information

S1 Results Table of manuscript dataset providing the mean, standard error of the mean (SEM) and p-values. (PDF)

## References

[1] Pope CN. Organophosphorus pesticides: do they all have the same mechanism of toxicity? J Toxicol Environ Health B Crit Rev. 1999 6;2(2):161–81. doi: 10.1080/109374099281205 10230392 10.1080/109374099281205

[2] Bouvier G, Blanchard O, Momas I, Seta N. Environmental and biological monitoring of exposure to organophosphorus pesticides: application to occupationally and non-occupationally exposed adult populations. J Expo Sci Environ Epidemiol. 2006 9;16(5):417–26. doi: 10.1038/sj.jes.7500473 16519410 10.1038/sj.jes.7500473

[3] Bolles HG, Dixon-White HE, Peterson RK, Tomerlin JR, Day EW, Oliver GR. U.S. market basket study to determine residues of the insecticide chlorpyrifos. J Agric Food Chem. 1999 5;47(5):1817–22. 10552457 10.1021/jf980962d

[4] Nougadère A, Sirot V, Kadar A, Fastier A, Truchot E, Vergnet C, et al Total diet study on pesticide residues in France: Levels in food as consumed and chronic dietary risk to consumers. Environ Int. 2012 9 15;45:135–50. doi: 10.1016/j.envint.2012.02.001 22595191 10.1016/j.envint.2012.02.001

[5] Lu C, Barr DB, Pearson MA, Waller LA. Dietary intake and its contribution to longitudinal organophosphorus pesticide exposure in urban/suburban children. Environ Health Perspect. 2008 4;116(4):537–42. doi: 10.1289/ehp.10912 18414640 10.1289/ehp.10912 PMC2290988

[6] Berton T, Mayhoub F, Chardon K, Duca R-C, Lestremau F, Bach V, et al Development of an analytical strategy based on LC-MS/MS for the measurement of different classes of pesticides and theirs metabolites in meconium: application and characterisation of foetal exposure in France. Environ Res. 2014 7;132:311–20. doi: 10.1016/j.envres.2014.03.034 24834827 10.1016/j.envres.2014.03.034

[7] Weldon RH, Barr DB, Trujillo C, Bradman A, Holland N, Eskenazi B. A pilot study of pesticides and PCBs in the breast milk of women residing in urban and agricultural communities of California. J Environ Monit JEM. 2011 11;13(11):3136–44. doi: 10.1039/c1em10469a 22009134 10.1039/c1em10469a

[8] Spyer KM, Gourine AV. Chemosensory pathways in the brainstem controlling cardiorespiratory activity. Philos Trans R Soc Lond B Biol Sci. 2009 9 12;364(1529):2603–10. doi: 10.1098/rstb.2009.0082 19651660 10.1098/rstb.2009.0082 PMC2865116

[9] Bartholomew PM, Gianutsos G, Cohen SD. Differential cholinesterase inhibition and muscarinic receptor changes in CD-1 mice made tolerant to malathion. Toxicol Appl Pharmacol. 1985 10;81(1):147–55. 4049416 10.1016/0041-008x(85)90129-2

[10] Carey JL, Dunn C, Gaspari RJ. Central respiratory failure during acute organophosphate poisoning. Respir Physiol Neurobiol. 2013 11 1;189(2):403–10. doi: 10.1016/j.resp.2013.07.022 23933009 10.1016/j.resp.2013.07.022

[11] Houze P, Pronzola L, Kayouka M, Villa A, Debray M, Baud FJ. Ventilatory effects of low-dose paraoxon result from central muscarinic effects. Toxicol Appl Pharmacol. 2008 12 1;233(2):186–92. doi: 10.1016/j.taap.2008.08.006 18775447 10.1016/j.taap.2008.08.006

[12] Villa AF, Houze P, Monier C, Risède P, Sarhan H, Borron SW, et al Toxic doses of paraoxon alter the respiratory pattern without causing respiratory failure in rats. Toxicology. 2007 3 22;232(1–2):37–49. doi: 10.1016/j.tox.2006.12.006 17250945 10.1016/j.tox.2006.12.006

[13] Chambers JE, Ma T, Boone JS, Chambers HW. Role of detoxication pathways in acute toxicity levels of phosphorothionate insecticides in the rat. Life Sci. 1994;54(18):1357–64. 7514706 10.1016/0024-3205(94)00515-x

[14] Moser VC, Chanda SM, Mortensen SR, Padilla S. Age- and gender-related differences in sensitivity to chlorpyrifos in the rat reflect developmental profiles of esterase activities. Toxicol Sci Off J Soc Toxicol. 1998 12;46(2):211–22. 10.1006/toxs.1998.2526 10048124

[15] Pope CN, Chakraborti TK, Chapman ML, Farrar JD, Arthun D. Comparison of in vivo cholinesterase inhibition in neonatal and adult rats by three organophosphorothioate insecticides. Toxicology. 1991;68(1):51–61. 1714639 10.1016/0300-483x(91)90061-5

[16] Whyatt RM, Rauh V, Barr DB, Camann DE, Andrews HF, Garfinkel R, et al Prenatal insecticide exposures and birth weight and length among an urban minority cohort. Environ Health Perspect. 2004 7;112(10):1125–32. doi: 10.1289/ehp.6641 15238288 10.1289/ehp.6641 PMC1247388

[17] Farag AT, El Okazy AM, El-Aswed AF. Developmental toxicity study of chlorpyrifos in rats. Reprod Toxicol Elmsford N. 2003 4;17(2):203–8. 10.1016/s0890-6238(02)00121-1 12642153

[18] Qiao D, Seidler FJ, Padilla S, Slotkin TA. Developmental neurotoxicity of chlorpyrifos: what is the vulnerable period? Environ Health Perspect. 2002 11;110(11):1097–103. 12417480 10.1289/ehp.021101097 PMC1241065

[19] Cochran RC, Kishiyama J, Aldous C, Carr WC, Pfeifer KF. Chlorpyrifos: hazard assessment based on a review of the effects of short-term and long-term exposure in animals and humans. Food Chem Toxicol Int J Publ Br Ind Biol Res Assoc. 1995 2;33(2):165–72. 10.1016/0278-6915(94)00124-7 7532610

[20] Drorbaugh JE, Fenn WO. A barometric method for measuring ventilation in newborn infants. Pediatrics. 1955 7;16(1):81–7. 14394741

[21] Christon J, Carley DW, Monti D, Radulovacki M. Effects of inspired gas on sleep-related apnea in the rat. J Appl Physiol Bethesda Md 1985. 1996 6;80(6):2102–7. 10.1152/jappl.1996.80.6.2102 8806919

[22] Ramadan W, Petitjean M, Loos N, Geloen A, Vardon G, Delanaud S, et al Effect of high-fat diet and metformin treatment on ventilation and sleep apnea in non-obese rats. Respir Physiol Neurobiol. 2006 1 25;150(1):52–65. doi: 10.1016/j.resp.2005.02.011 16448934 10.1016/j.resp.2005.02.011

[23] McGuire M, MacDermott M. The influence of streptozotocin-induced diabetes and the antihyperglycaemic agent metformin on the contractile characteristics and the membrane potential of the rat diaphragm. Exp Physiol. 1998 7;83(4):481–7. 9717070 10.1113/expphysiol.1998.sp004131

[24] Ellman GL, Courtney KD, Andres V, Feather-Stone RM. A new and rapid colorimetric determination of acetylcholinesterase activity. Biochem Pharmacol. 1961 7;7:88–95. 13726518 10.1016/0006-2952(61)90145-9

[25] Perera FP, Rauh V, Tsai W-Y, Kinney P, Camann D, Barr D, et al Effects of transplacental exposure to environmental pollutants on birth outcomes in a multiethnic population. Environ Health Perspect. 2003 2;111(2):201–5. 12573906 10.1289/ehp.5742 PMC1241351

[26] Curzi-Dascalova L, Christova-Guéorguiéva E. Respiratory pauses in normal prematurely born infants. A comparison with full-term newborns. Biol Neonate. 1983;44(6):325–32. 6686064 10.1159/000241747

[27] Bird SB, Gaspari RJ, Dickson EW. Early death due to severe organophosphate poisoning is a centrally mediated process. Acad Emerg Med Off J Soc Acad Emerg Med. 2003 4;10(4):295–8. 10.1111/j.1553-2712.2003.tb01338.x 12670839

[28] Tang J, Carr RL, Chambers JE. Changes in rat brain cholinesterase activity and muscarinic receptor density during and after repeated oral exposure to chlorpyrifos in early postnatal development. Toxicol Sci Off J Soc Toxicol. 1999 10;51(2):265–72. 10.1093/toxsci/51.2.265 10543028

[29] Slotkin TA, Seidler FJ. Comparative developmental neurotoxicity of organophosphates in vivo: transcriptional responses of pathways for brain cell development, cell signaling, cytotoxicity and neurotransmitter systems. Brain Res Bull. 2007 5 30;72(4–6):232–74. doi: 10.1016/j.brainresbull.2007.01.005 17452286 10.1016/j.brainresbull.2007.01.005 PMC1945108

[30] Rauh VA, Garfinkel R, Perera FP, Andrews HF, Hoepner L, Barr DB, et al Impact of prenatal chlorpyrifos exposure on neurodevelopment in the first 3 years of life among inner-city children. Pediatrics. 2006 12;118(6):e1845–1859. doi: 10.1542/peds.2006-0338 17116700 10.1542/peds.2006-0338 PMC3390915

[31] Chambers JE, Chambers HW. Oxidative desulfuration of chlorpyrifos, chlorpyrifos-methyl, and leptophos by rat brain and liver. J Biochem Toxicol. 1989;4(3):201–3. 2481746 10.1002/jbt.2570040310

[32] Proskocil BJ, Bruun DA, Thompson CM, Fryer AD, Lein PJ. Organophosphorus pesticides decrease M2 muscarinic receptor function in guinea pig airway nerves via indirect mechanisms. PloS One. 2010;5(5):e10562 doi: 10.1371/journal.pone.0010562 20479945 10.1371/journal.pone.0010562 PMC2866713

[33] Mendelson WB, Martin JV, Perlis M, Giesen H, Wagner R, Rapoport SI. Periodic cessation of respiratory effort during sleep in adult rats. Physiol Behav. 1988;43(2):229–34. 3212061 10.1016/0031-9384(88)90243-0

[34] Gaspari RJ, Paydarfar D. Dichlorvos-induced central apnea: effects of selective brainstem exposure in the rat. Neurotoxicology. 2011 3;32(2):206–14. doi: 10.1016/j.neuro.2011.01.005 21241738 10.1016/j.neuro.2011.01.005 PMC3063523

[35] Gillis RA, Walton DP, Quest JA, Namath IJ, Hamosh P, Dretchen KL. Cardiorespiratory effects produced by activation of cholinergic muscarinic receptors on the ventral surface of the medulla. J Pharmacol Exp Ther. 1988 11;247(2):765–73. 3183970

[36] Segura P, Chávez J, Montaño LM, Vargas MH, Delaunois A, Carbajal V, et al Identification of mechanisms involved in the acute airway toxicity induced by parathion. Naunyn Schmiedebergs Arch Pharmacol. 1999 12;360(6):699–710. 10619188 10.1007/s002109900101

[37] Hara Y, Noda A, Miyata S, Otake H, Yasuda Y, Okuda M, et al Comparison of oxygen desaturation patterns in children and adults with sleep-disordered breathing. Am J Otolaryngol. 2013 10;34(5):537–40. doi: 10.1016/j.amjoto.2013.01.014 23453118 10.1016/j.amjoto.2013.01.014

[38] Burd PF, Ferry CB. A prolonged contraction at the end-plate region of the diaphragm of rats and mice after anticholinesterases in vitro. J Physiol. 1987 10;391:429–40. 3443954 10.1113/jphysiol.1987.sp016747 PMC1192223

[39] Wright PG. An analysis of the central and peripheral components of respiratory failure produced by anticholinesterase poisoning in the rabbit. J Physiol. 1954 10 28;126(1):52–70. 13212728 10.1113/jphysiol.1954.sp005191 PMC1365640

[40] Hulse EJ, Davies JOJ, Simpson AJ, Sciuto AM, Eddleston M. Respiratory complications of organophosphorus nerve agent and insecticide poisoning. Implications for respiratory and critical care. Am J Respir Crit Care Med. 2014 12 15;190(12):1342–54. doi: 10.1164/rccm.201406-1150CI 25419614 10.1164/rccm.201406-1150CI PMC4299648

