# Supplementary material for: Impact of chronic exposure to the pesticide chlorpyrifos on respiratory parameters and sleep apnea in juvenile and adult rats
Source: PLoS One. 2018 Jan 22;13(1):e0191237. doi: 10.1371/journal.pone.0191237 (PMC5777649; doi:10.1371/journal.pone.0191237)
Supplement: S1 Results — (PDF) [file pone.0191237.s001.pdf]

Figure 1: Body weight

	Control		CPF1		CPF5	
	Mean	SEM	Mean	SEM	Mean	SEM
PND01	6,714	0,1451	6,122	0,09	5,667	0,2757
PND21	52,4	1,4	44,63	0,38	47,63	2,05
PND60	331,7	7,039	294,4	6,2	284,3	10,4

Two-way ANOVA		
	P value	
Interaction	< 0.0001	***
CPF exposui	< 0.0001	***
age	< 0.0001	***

Figure 2: Respiration

	Control		CPF1		CPF5	
Figure 2A: T_I (	Mean	SEM	Mean	SEM	Mean	SEM
PND21	174,501	17,0914	176,531	8,2254	181,202	9,6405
PND60	214,361	20,2177	222,117	18,1805	221,277	26,594

	P value	
Interaction	0,950	ns
CPF exposui	0,649	ns
age	0,005	**

	Control		CPF1		CPF5	
Figure 2B: T_E (	Mean	SEM	Mean	SEM	Mean	SEM
PND21	250,00	18,87	253,70	11,54	267,46	16,96
PND60	323,96	24,62	366,42	36,39	373,59	21,23

	P value	
Interaction	0,195	ns
CPF exposui	0,012	*
age	< 0.0001	***

	P value
CPF-1 vs Control	0,011
CPF-5 vs Control	0,0006

	Control		CPF1		CPF5	
Figure 2C: f (b	Mean	SEM	Mean	SEM	Mean	SEM
PND21	156,08	8,66	156,46	6,58	159,63	11,19
PND60	135,20	12,64	113,63	5,47	104,21	2,00

	P value	
Interaction	0,0712	ns
CPF exposui	0,2177	ns
age	< 0.0001	***

PND60	P value
CPF-5 vs Control	0,04

	Control		CPF1		CPF5	
Figure 2D: V_T (	Mean	SEM	Mean	SEM	Mean	SEM
PND21	0,96	0,23	0,76	0,06	0,69	0,06
PND60	0,39	0,04	0,51	0,05	0,49	0,10

	P value	
Interaction	< 0.0001	***
CPF exposui	0,164	ns
age	< 0.0001	***

PND21	P value
CPF-1 vs Control	0,0586
CPF-5 vs Control	0,0153

PND60	P value
CPF-1 vs Cont	0,0004
CPF-5 vs Cont	0,044

Figure 3: Sleep apnea index

	Control		CPF1		CPF5	
Figure 3B: Sle	Mean	SEM	Mean	SEM	Mean	SEM
PND21	16,57	2,79	20,11	1,28	18,69	1,04
PND60	11,63	4,26	19,49	4,24	23,77	4,43

	P value	
Interaction	0,014	*
CPF exposui	0,0002	***
age	0,909	ns

	P value
CPF-1 vs Control	0,005
CPF-5 vs Control	0,002

	P value
Control: PND21 vs PND	0,081
CPF-5: PND21 vs PND6	0,03

Figure 4: Diaphragm contractility

	Control		CPF1		CPF5	
Figure 4A: Tw	Mean	SEM	Mean	SEM	Mean	SEM
PND21	49,26	9,94	77,21	14,05	99,65	21,98
PND60	39,33	11,31	62,51	11,66	119,15	14,21

	P value	
Interaction	0,422	ns
CPF exposui	< 0.0001	***
age	0,882	ns

	P value
CPF-1 vs Control	0,102
CPF-5 vs Control	<0,0001
CPF-1 vs CPF-5	0,0045

	Control		CPF1		CPF5	
Figure 4B: Tin	Mean	SEM	Mean	SEM	Mean	SEM
PND21	20,51	0,87	21,11	0,47	22,60	0,80
PND60	15,42	0,59	15,04	0,28	15,63	0,57

	P value	
Interaction	0,353	ns
CPF exposui	0,156	ns
age	< 0.0001	***

	Control		CPF1		CPF5	
Figure 4C: Hal	Mean	SEM	Mean	SEM	Mean	SEM
PND21	19,91	0,46	20,51	0,69	19,83	0,96
PND60	17,38	1,74	15,55	0,94	15,88	0,86

	P value	
Interaction	0,449	ns
CPF exposui	0,686	ns
age	< 0.0001	***

	Control		CPF1		CPF5	
Figure 4D: Fat	Mean	SEM	Mean	SEM	Mean	SEM
PND21	54,71	4,38	54,61	2,74	59,86	4,48
PND60	67,85	4,33	73,35	2,51	80,09	2,07

	P value	
Interaction	0,579	ns
CPF exposui	0,051	ns
age	< 0.0001	***

	P value
CPF-5 vs Control	0,004
CPF-1 vs CPF-5	0,067

Figure 5: AChE activity

	Control		CPF1		CPF5	
AChE activity	Mean	SEM	Mean	SEM	Mean	SEM
PND21	0,027	0,002	0,021	0,002	0,014	0,002
PND60	0,024	0,001	0,026	0,002	0,019	0,001

	P value	
Interaction	0,074	ns
CPF exposui	< 0.0001	***
age	0,272	ns

	P value
CPF-5 vs Control	<0,001
CPF-1 vs CPF-5	0,002
